# Volition and Perception: Why patients choose to continue or discontinue ADHD medication

**DOI:** 10.1192/j.eurpsy.2024.424

**Published:** 2024-08-27

**Authors:** K. B. Johannessen, P. Ishøy, T. Houmann, E. Levin, P. H. Thomsen

**Affiliations:** ^1^Psychiatric Center Glostrup, Brøndby; ^2^Psychiatric Center Hillerød, Hillerød; ^3^Aarhus University, Aarhus, Denmark

## Abstract

**Introduction:**

The present study examines self-reported factors related to discontinuation of ADHD medication in Danish adults. Based on insights from six patient interviews, a questionnaire was developed with themes such as perception of ADHD, perceived beneficial- and adverse effects of the medication, to examine patients’ reasons for continuation or discontinuation of the prescribed medication.

**Objectives:**

ADHD medication has proven effective for treating ADHD in adults. Large registry-based studies have generally shown high discontinuation rates over time and focused on different risk factors, such as comorbidity, gender and socioeconomic status. However, in the present study we explore patient reported reasons for continuation or discontinuation of ADHD medication as well as what drives their choice of living with or without medication despite ADHD.

**Methods:**

The present research is a questionnaire study consisting of 1,050 Danish adults who redeemed a prescription of ADHD medication for the first time between 2017-2019. Questionnaires were sent out by Statistics Denmark to 4.748 adults, a representative sample from the 17.334 Danish adults who redeemed a prescription within that period. A gap of 12 months between redemptions was defined as discontinuation and questionnaires were sent out to an equal number of patients who continued or discontinued the ADHD-medication. Chi^2^-tests were performed to examine the differences between adults who continued vs. discontinued ADHD-medication in relation to different main themes.

**Results:**

The patients who continued medical treatment more strongly perceived ADHD as a biological illness whereas patients who discontinued, more strongly perceived ADHD as an illness constructed by society. Furthermore, patients who continued medical treatment reported that the medication has a more positive influence on their lives whereas patients who discontinued the medication reported that the treatment involved more negative feelings and decreased the positive sides of themselves. Finally, patients who continued the prescribed ADHD-drugs reported more strongly that they continued the treatment for themselves, to be able to work and be social than the patients who discontinued the medical treatment.

**Conclusions:**

The present findings suggest that the perception of ADHD as being either a biological or social construct is central to why patients choose to continue or discontinue ADHD-medication. Moreover, patients who continued the medical treatment generally reported more positive effects of the ADHD-drugs whereas patients who discontinued the medical treatment reported different negative effects of the medication. From a clinical perspective, these findings show the importance of understanding the individual patient’s perception of ADHD. These perspectives should be addressed in the clinic alongside with awareness of how ADHD-drugs may have a positive and negative effect on the individual patient.

**Disclosure of Interest:**

None Declared

